# Operando Benchtop NMR Quantifies Carbonation, Water Crossover, and Liquid Products for High-Current Electrochemical CO_2_ Reduction

**DOI:** 10.1021/acscatal.5c00355

**Published:** 2025-07-07

**Authors:** Zhiyu Zhu, Kaan Zeki Çolakhasanoĝlu, Ruud L.E.G. Aspers, Joris Meurs, Simona M. Cristescu, Thomas Burdyny, Evan Wenbo Zhao

**Affiliations:** † Magnetic Resonance Research Center, Institute for Molecules and Materials, 6029 Radboud University, Nijmegen 6525 AJ, The Netherlands; ‡ Life Science Trace Detection Laboratory, Institute for Molecules and Materials, 98810 Radboud University, Nijmegen 6525 AJ, The Netherlands; § Department of Chemical Engineering, 2860 Delft University of Technology, Delft 2629 HZ, The Netherlands

**Keywords:** electrochemical CO_2_ reduction, solution NMR, electrocatalysis, operando NMR, in situ NMR

## Abstract

Operando characterization is crucial for understanding the selectivity and stability of the electrochemical CO_2_ reduction reaction (eCO_2_RR). Existing operando techniques normally use single-compartment cells operating at low currents. However, high current densities on the order of 100 mA cm^–2^ are required for practical applications. Under a high current, reaction pathways and electrolyte dynamics can change, and stability issues such as salt precipitation and water crossover become more pronounced. Here, we developed an inline operando NMR method that is compatible with high-current reaction conditions. Demonstrating this on a copper-catalyzed eCO_2_RR at 100 mA cm^–2^, the operando NMR revealed a fast decrease of Faradaic efficiency for formate and ethanol within half an hour of reaction, accompanied by a pH decrease from 14 to 8 and a continuous accumulation of bicarbonate in the electrolyte. Water crossover was simultaneously observed and quantified via a deuteration technique and became more severe at high currents. This study revealed a highly dynamic electrolyte environment of copper-catalyzed eCO_2_RR. Using a gas diffusion flow cell and a benchtop NMR system, this operando approach is accessible by non-NMR experts and readily applicable to a wide range of catalysts, electrolyte compositions, and reactor designs for eCO_2_RR.

## Graphical abstract

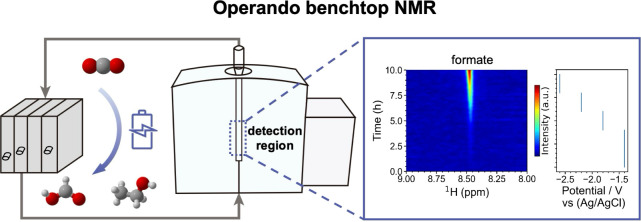

## Introduction

Electrochemical conversion of CO_2_ into value-added chemicals has been widely accepted as a promising approach to address the CO_2_ challenge. However, issues of low selectivity and stability have hindered a widespread application in industry.^1,2^ Improving the selectivity and stability requires an understanding of reaction and degradation mechanisms at the molecular and device levels. Here, operando characterization techniques, particularly under realistic reaction conditions, can be extremely useful.^3,4^ Operando refers to the mode of measurement performed during an electrochemical reaction. Because applied electrical potentials influence thermodynamic and kinetic pathways, postmortem analysis could result in a misleading understanding due to relaxation effects or external sample contaminations. By contrast, operando characterization minimizes the relaxation effect and bypasses artificial errors, allowing the most reliable measure of reaction kinetics and detecting short-lived intermediates.

A number of operando methods have been demonstrated for studying electrochemical CO_2_ reduction reaction (eCO_2_RR), chromatography, and mass spectrometry (MS) being the prevalent ones.^5–11^ However, both techniques require specialized in situ cells that are commonly adapted from a single-compartment cell, limiting the current density to a few mA cm^–2^ due to the sluggish mass transport.^5,7,11^ For practical applications, high current density on the order of hundreds of mA cm^–2^ is required. Thus, the reaction has been mostly conducted in a flow cell with gas diffusion electrodes.^12,13^ Although inline measurements of gas products by gas chromatography have been performed during eCO_2_RR, liquid products such as formate and ethanol have been harder to quantify in real time and instead require aliquoting and ex situ measurements, which typically result in unlinked gas–liquid product Faradaic efficiencies.

A further challenge of analyzing electrochemical cells is the imbalanced catholyte and anolyte conditions that exist during long-term operations. For example, previous work has shown that the pH and carbon balance are affected depending on the membrane type,^14^ while species crossing the membrane can also be oxidized at the anode. An operando technique to study the electrolyte environment at a high current density can further help elucidate the scales and mechanisms of the numerous transport phenomena.

Nuclear magnetic resonance (NMR) is a noninvasive, element-specific, and quantitative technique. Operando/in situ electrochemical NMR was first demonstrated by Richards and Evans in 1975 and further developed and optimized by other groups.^15–22^ Typically, two or three electrodes were inserted into a standard 5- or 10-mm NMR tube, and the tube is converted into a single-compartment electrochemical cell. While it has been shown to be informative for studying the local pH^17,19^ and investigating ion exchange and reaction mechanisms for eCO_2_RR,^16–20^ the applied current density is on the order of a few mA cm^–2^ or less, limited by the mass transport inside a single-compartment cell and thus the measurement condition do not fully reflect realistic electrolysis conditions.

Inspired by the concept of spatially separating the electrochemistry operation from the NMR detection,^22^ we present here a new operando NMR method for studying eCO_2_RR under high-current density, using a benchtop NMR coupled with a gas diffusion electrolyzer. Minimum modification is required to integrate the reactor into the measurement modality since NMR and electrochemical cell can be connected via flow, making the measurement straightforward to set up. The NMR spectroscopic resolution is maximized because only a flowing solution is present in the detection region, eliminating the influence of electrodes on NMR detection. Furthermore, we performed the study on a benchtop system, significantly increasing the accessibility of this operando technique.

The operando study was performed on copper-catalyzed eCO_2_RR, with copper being one of the most used catalysts capable of producing C_2_^+^ products. We quantified the liquid products as a function of potential and time and observed a decrease in the Faradaic efficiencies of formate and ethanol as the copper electrode’s selectivity degraded. In the bulk electrolyte, chemical shift of water was used as an indicator to capture the carbonation of the electrolyte solution and quantify the (bi)­carbonate concentration. The concentration increases during the reaction and have a strong potential dependence. Finally, we observed and quantified water crossover as a function of time and current densities, and the strong current dependence provides evidence that water molecules transport through the membranes by solvating the charge-balancing ion. The water and ion imbalance together led to the failure of the electrolyzer.

## Results and Discussion

The results and discussion section is organized into four subsections. Developing an Operando Benchtop NMR Method describes the operando NMR method, followed by Time-Resolved Quantification of Liquid Products and Faradaic Efficiencies, which focuses on quantifying liquid products, specifically formate and ethanol. Capturing Electrolyte Carbonation discusses on quantifying carbonates, while Monitoring Water Crossover covers water crossover.

### Developing an Operando Benchtop NMR Method

An eCO_2_RR reactor system consists of two electrolyte reservoirs, a CO_2_ gas source, and a gas diffusion flow cell, as schematically illustrated in Figure 1. The electrolyte solutions flow from the catholyte and anolyte reservoirs, respectively, through the flow cell, where the reactions occur. Within the flow cell, a gas diffusion layer is placed in contact with the cathode to feed CO_2_ gas to the Cu catalysts. A back-pressure regulator is installed on the gas line to balance the gas and liquid pressures from both sides of the gas diffusion electrode, preventing flooding of the gas compartment. CO_2_ is reduced on the cathode, and water is oxidized on the anode.

**Figure 1:**
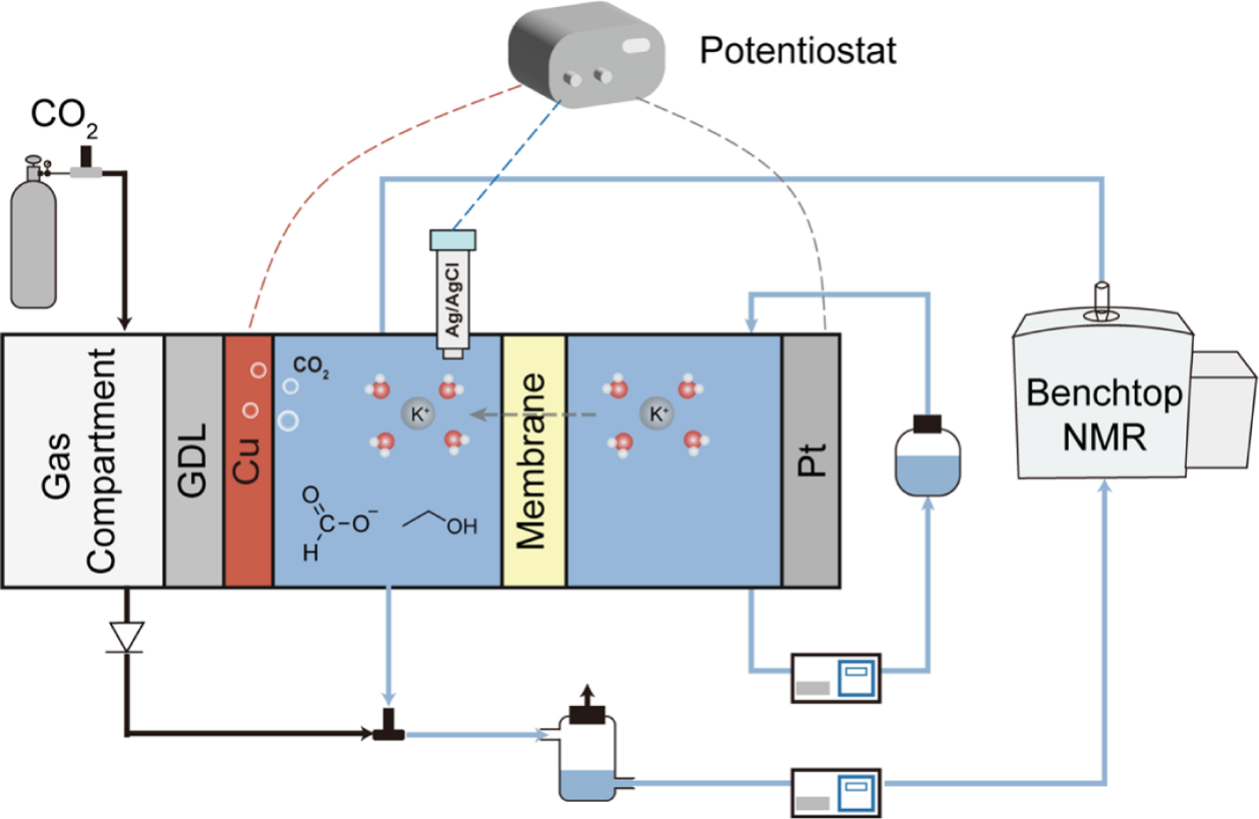
Schematic of the inline operando benchtop NMR for studying eCO_2_RR. The flow cell comprises a gas compartment, gas diffusion layer (GDL), Cu working electrode, catholyte compartment, cation exchange membrane, anolyte compartment, and Pt counter electrode, respectively. A reference electrode, Ag/AgCl, was inserted into the catholyte compartment. The volume of the tubes and NMR probe is approximately 5.8 mL. At a flow rate of 2.5 mL/min, the time delay between the electrochemical conversion and NMR measurement is 2.3 min.

The electrolyte flow is leveraged to couple the reactor to benchtop NMR, for which a Fourier 80 system is employed. A flow-through NMR tube was applied to perform the NMR measurement: the catholyte solution flows from a reservoir into the NMR detection region and then back to the electrolyte reservoir. As gas bubbles are undesirable to NMR detection due to their downgrading of magnetic field homogeneity, gas bubbles were removed via an opening in the cap of the catholyte reservoir. Compared to other operando NMR methods where a miniturized electrochemical cell is inserted into the detection region,^15–21,23,24^ our inline configuration allows the independent optimization of the electrochemical performance and NMR measurement, achieving high-rate eCO_2_RR and the most sensitive NMR detection on a given spectrometer. Thus, sophisticated electrochemical cells can be used with little to no modifications.

### Time-Resolved Quantification of Liquid Products and Faradaic Efficiencies

Formate and ethanol are the primary liquid products of the eCO_2_RR. Here time-dependent evolution of these species was evaluated using the operando NMR method.

First, chronoamperometry was performed from −1.4 to −2.6 V (versus Ag/AgCl) in decremental steps of 0.4 V. Cu mesh, Ag/AgCl, and Pt foils are utilized as the working, reference, and counter electrode, respectively. As shown in Figure 2a, at a potential of −1.4 V, the current stabilized at 3.5 mA cm^–2^. While the current density remained relatively stable between 32 and 37 mA cm^–2^ at −1.8 V, it increased from 85 to 115 mA cm^–2^ at −2.2 V and from 190 to 250 mA cm^–2^ at −2.4 V. The operando ^1^H NMR spectra of formate are shown on the left of Figure 2a. The formate ^1^H resonance at 8.45 ppm started to become visible at −1.4 V (a stack plot is shown in Figure S2), and the signal grew slowly but remained barely visible at −1.4 and −1.8 V. Once the potential was decreased to–2.2 V, the formate signal started to increase at a significantly faster rate.

**Figure 2:**
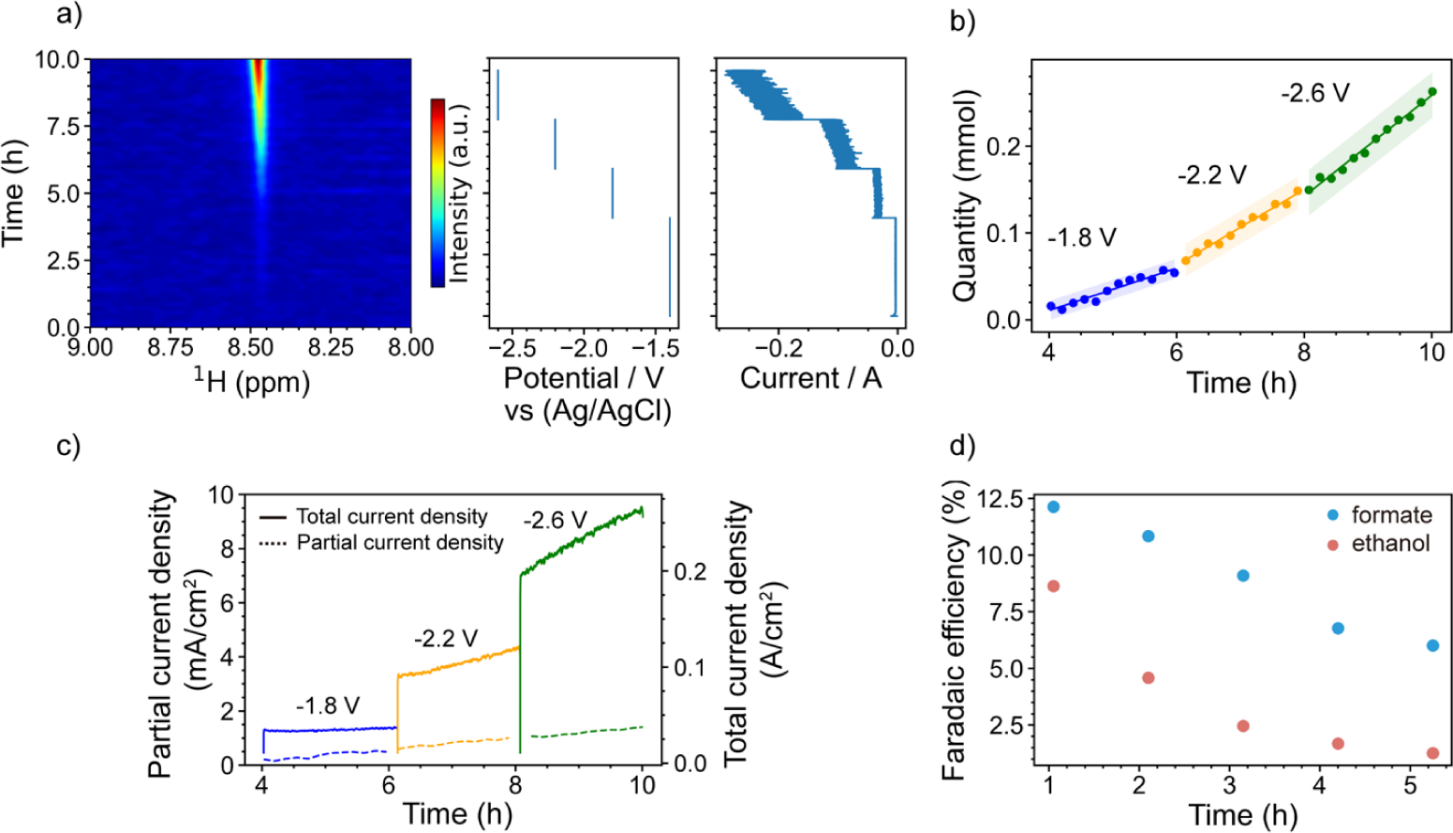
(a) Operando ^1^H NMR showing the evolution of the formate resonances as a function of time (left) and the corresponding potential and current profiles (right). (b) Quantity of formate and fitted overall reaction rate at different potentials. The shaded region is the 95% confidence interval. (c) The partial current density and total current density at different potentials. The solid line represents total current density (right axis), while the dashed line represents partial current density (left axis). (d) FE values of formate and ethanol as a function of time. The corresponding operando NMR spectra are shown in Figure S4. Note that data in (a–c) were acquired on a Cu mesh catalyst, and data in (d) were acquired on a sputtered Cu catalyst in order to increase the selectivity for ethanol.

Formate was quantified via a calibration curve (Figure S1b). As shown in Figure 2b, the amount increased from 0.016 to 0.068 mmol at −1.8 V, from 0.068 to 0.15 mmol at −2.2 V, and from 0.15 to 0.26 mmol at −2.6 V. The total average Faradaic efficiency is shown in Table S1. (The detailed calculation can be found in the *Operando NMR Quantification of Formate* section of the Supporting Information.) The quantity of formate was then converted to the partial current density as a function of time at different applied potentials, as shown in Figure 2c. The overall reaction rate was 0.026, 0.041, and 0.055 mmol/h at −1.8, −2.2, and −2.6 V, respectively. The highest FE is achieved at −1.4 V, where the total current density is the lowest, in agreement with previous findings that the FE of formate decreases with increasing current density.^12^

In a brief summary, formate has been successfully quantified during the course of the reaction via an operando NMR technique. Such a technique can be readily extended to track formate production with Ag or post-transition metals such as Sn by changing the catalyst on a polytetrafluoroethylene membrane.

Ethanol was monitored by performing chronopotentiometry at 100 mA cm^–2^ while ^1^H NMR spectra were acquired. Sputtered Cu nanoparticles, Ag/AgCl, and Pt foils were used as the working, reference, and counter electrode, respectively. The ethanol and formate concentrations were quantified (calibration curve is shown in Figure S6 and Tables S2 and S3). The FE as a function of time was calculated, as shown in Figure 2d (details in the *Quantification of C*_*2*_ *^+^liquid products* section of the Supporting Information). The FE of formate decreased from 12.13% to 6.01%, and the FE of ethanol decreased from 8.64% to 1.27%. Besides the possible reconstruction of Cu catalysts,^25,26^ we hypothesize that CO_2_ initially reacts with KOH to form KHCO_3_, leading to the low FE of ethanol, echoing the literature, the conductivity of KHCO_3_ is lower than KOH, negatively affecting the reaction rate and selectivity.^12,27,28^ This carbonation process is investigated further and reported in the following section.

### Capturing Electrolyte Carbonation

The pH equilibrium, along with the carbonate and bicarbonate equilibrium, significantly affects the stability and selectivity.^29,30^ These equilibria are coupled and involve both chemical and electrochemical reactions, as described by the reactions below:

1 $${\mathrm{2OH}}^{-}{+\mathrm{CO}}_{2}⇌{\mathrm{CO}}_{3}^{2-}+H_{2}O$$

2 $${\mathrm{CO}}_{3}^{2-}{+\mathrm{CO}}_{2}{+H}_{2}O⇌{{\mathrm{2HCO}}_{3}}^{-}$$

3 $${2H}_{2}{O+2e}^{-}\to H_{2}{+\mathrm{2OH}}^{-}$$

4 $${\mathrm{CO}}_{2}{+H}_{2}{O+\mathrm{ne}}^{-}\to {\mathrm{Product}+\mathrm{OH}}^{-}$$

Chemical shift of water is sensitive to the pH and the electrolyte environment. Because of the exchange between OH^–^ and H_2_O, the chemical shift becomes a function of OH^–^ concentration.^31^ Concentrations of HCO_3_^–^ and CO_3_^2–^ also influence the chemical shift due to their ionic charge effects and impact on water cluster size.^32^ The chemical shift of water provides a measure of the pH and (bi)­carbonate concentrations.

Figure 3 presents the ^1^H NMR spectra of water as a function of time at increasing currents during the eCO_2_RR. The measured potential for each spectrum is presented in Figure S8. Without a CO_2_ flow and an electrical current, the water resonance remains at 4.90 ppm (Figure 3a). Once the CO_2_ flow is on, the water resonance shifts from 4.90 to 4.80 ppm within the first 0.5 h, then remains stable at 4.80 ppm (Figure 3b). When the current is set at 50 mA cm^–2^, as the CO_2_ reduction progresses, the water resonance shifts from 4.90 to 4.80 ppm, then to 4.82 ppm (Figure 3c), with the shifts becoming more pronounced at higher currents (Figure 3d–f).

**Figure 3:**
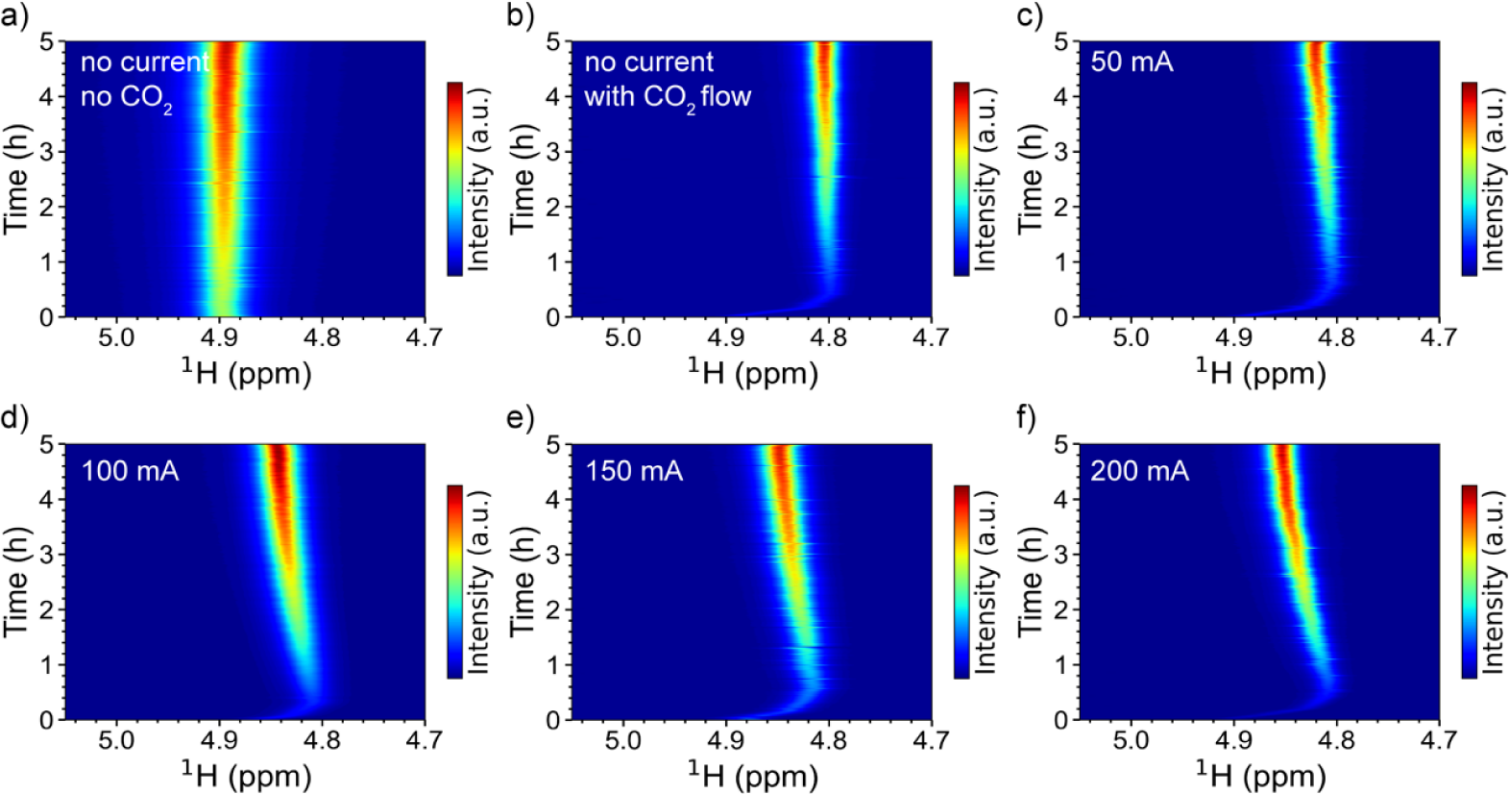
(a, b) Pseudo-2D ^1^H NMR spectra of water as a function of time with and without a CO_2_ flow. (c–f) ^1^H NMR spectra of water at different currents. Flow rates of CO_2_ and electrolyte solution are 10 and 2.5 mL/min, respectively. Electrode nominal area is 1 cm^2^. In the anolyte, 1 M KOH was dissolved in H_2_O; 1 M KOH was dissolved in D_2_O in the catholyte. Sputtered Cu, Ag/AgCl, and Pt foils were used as the working, reference, and counter electrodes, respectively.

The shift of water resonance without any current is caused by a combined effect of pH, HCO_3_^–^, and CO_3_^2–^ anions, following Reactions 1 and 2, which increase the acidity of the electrolyte solution and the concentrations of HCO_3_^–^ and CO_3_^2–^ anions. The increase in the concentrations of HCO_3_^–^ and CO_3_^2–^ causes the chemical shift of water to increase, while higher acidity leads to a decrease in the chemical shift. Since the latter was observed within the first 0.5 h of the experiments with and without currents (Figure 3b–f), the pH effect is dominant within the first 0.5 h. To verify the pH effect, pH values were measured for two experiments with and without currents, and the results are shown in Figure 4a,b. In both experiments, the pH values decrease drastically from 14 to 7.8 within the first 0.5 h upon turning on the CO_2_ flow, then stabilize at around 8. The pH decrease correlates strongly with the shift of water resonance, confirming the dominant effect of pH on the chemical shift of water during the dissolution of CO_2_ into the electrolyte solution.

**Figure 4:**
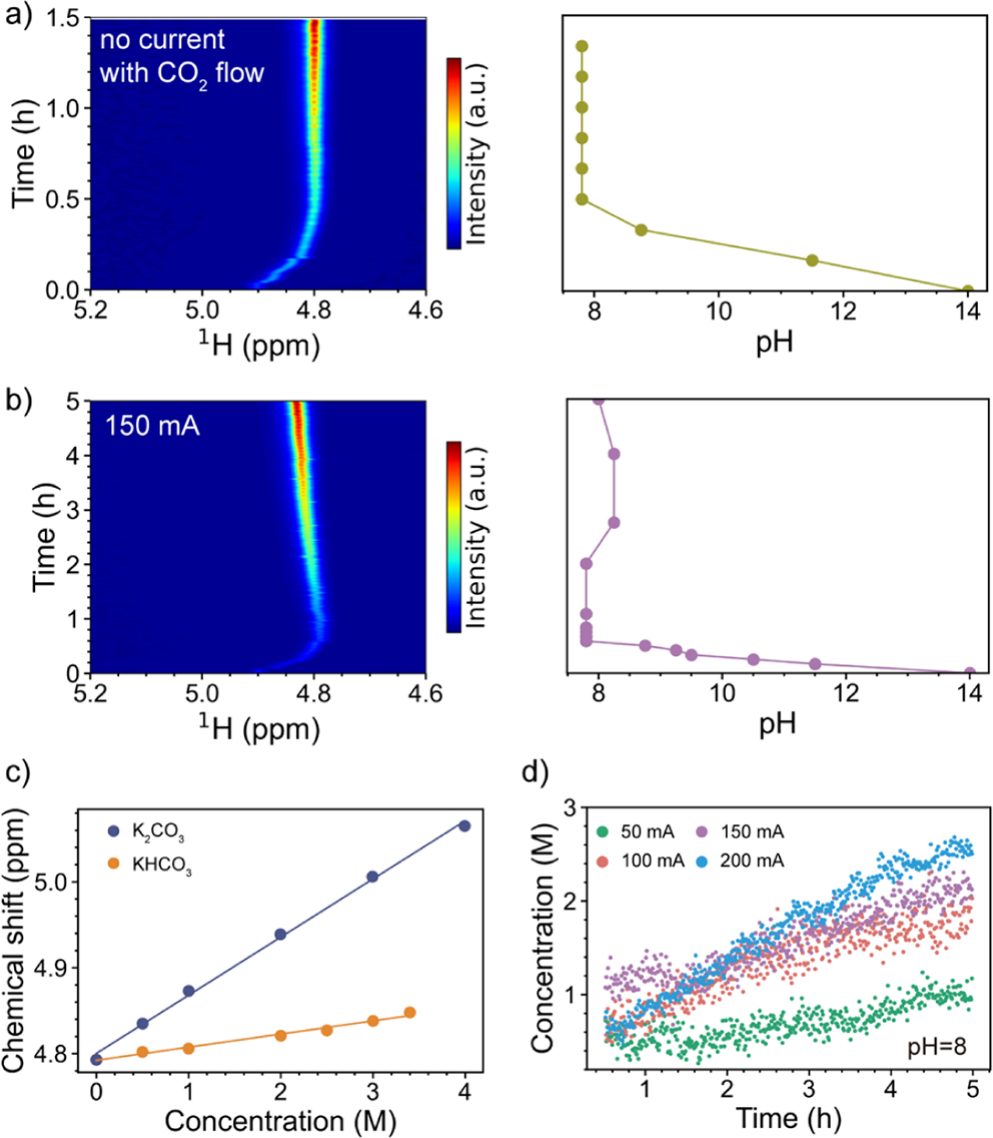
(a, b) Pseudo-2D ^1^H NMR spectra of water and pH values of the electrolyte solution as a function of time. The pH values were measured by pH paper during the reaction (see details in Figure S9). (c) Chemical shift of water as a function of KHCO_3_ and K_2_CO_3_ concentrations. (d) NMR-derived HCO_3_^–^ concentrations at different currents at a pH of 8.

Chronopotentiometry was performed from 0 to 200 mA cm^–2^. At high current densities of 100, 150, and 200 mA cm^–2^, water resonance shifted toward a higher chemical shift. Since pH barely changed during the reaction, as shown in Figure 4b, this shift is attributed to the concentration change of HCO_3_^–^ and CO_3_^2–^ anions.^32^ Following Reactions 1–4, both H_2_O and CO_2_ reduction produce OH^–^ anions, which further react with CO_2_ to form HCO_3_^–^ and CO_3_^2–^. Thus, the water resonance started to shift toward a higher chemical shift after 0.5 h. HCO_3_^–^ and CO_3_^2–^ concentration increases at higher current density, leading to a more pronounced shift of the water resonance. To quantify the ionic charge effect, we measured the chemical shift of water resonance as a function of KHCO_3_ and K_2_CO_3_ concentrations, as shown in Figure 4c. The chemical shift increases linearly as a function of the KHCO_3_ and K_2_CO_3_ concentrations, respectively.

Since the equilibrium between HCO_3_^–^ and CO_3_^2–^ anions in the electrolyte solution is known with a p*K*_a_ of 10.3,^17^ the concentration of (bi)­carbonate can be estimated. Based on the measured pH and taking into account the errors, we calculated the concentration within a pH range between 7.5 and 9.5 (see *Quantifying time-resolved bicarbonate concentration* section of the Supporting Information). The concentration of HCO_3_^–^ shows a negligible pH dependence within this range, as the solution remains predominantly HCO_3_^–^. The calculated HCO_3_^–^ concentrations at different currents and pH of 8 are presented in Figure 4d. At higher current density, the bicarbonate concentration increased more significantly due to more OH^–^ being generated based on Reactions 3 and 4. At 200 mA cm^–2^, the concentration of bicarbonate reached 2.8 M in the bulk electrolyte. The higher carbonation rate of the electrolyte at higher current densities will likely lead to faster salt precipitation and shorter lifetime of the electrolysis.

### Monitoring Water Crossover

In the previous section on ethanol quantification, we observed water crossover through visual inspection of the liquid level in the electrolyte reservoir. To monitor and quantify water crossover at the molecular level, deuterium-labeled D_2_O was used for the catholyte and H_2_O for the anolyte, respectively. Operando ^1^H NMR measurements were performed on the catholyte solution as a function of current, and the results are shown in Figures 3 and S8. In all experiments, the intensity of the H_2_O signal increases during the reaction. By integration of the signal, the fraction of H_2_O is quantified as presented in Figure 5a,b.

**Figure 5:**
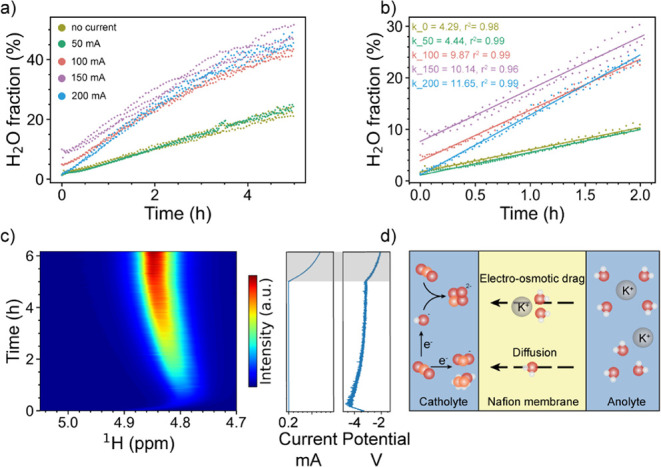
(a) H_2_O fraction as a function of time at different currents. The starting point differs slightly due to residual H_2_O in the tubing. When the H_2_O fraction increases, the proton signal becomes notably scattered, possibly due to the influence of the pulsating flow driven by the peristaltic pump. (b) Fitted data at different current densities. *k* represents the slope, i.e., the water crossover rate. (c) Pseudo-2D ^1^H NMR spectra of water as a function of time at 200 mA cm^–2^ (left) and the corresponding current and potential of the electrolyzer (right). The shaded region highlights the period after the overload of the potentiostat. (d) Schematic of the electro-osmotic drag and diffusion of water molecules through the membrane. Red atom: oxygen, orange: carbon, white: hydrogen.

The H_2_O fraction as a function of time was linear for the first 2 h, and then the slope gradually decreased. This phenomenon comes from two crossover processes occurring simultaneously in the system, as schematically illustrated in Figure 5d. One process is driven by electro-osmosis, and the other by diffusion, with the former being concentration-independent and the latter concentration-dependent. After 2 h, the H_2_O fraction on the cathode side significantly increased, and the exchange of D_2_O and H_2_O slowed down as the concentration difference became smaller, reducing the driving force for diffusion. To obtain the crossover rate driven by the current density, we choose the linear region (first 2 h of data) for fitting where the diffusion-driven crossover rate is also linear to a first approximation. The fitted data at different current densities are presented in Figure 5b.

The water crossover rate from 0 to 200 mA cm^–2^ is 0.45, 0.47, 1.10, 1.23, and 1.32 mL/h, respectively. At 50 mA cm^–2^, the crossover rate is close to that observed with no current, indicating that at low current densities, water crossover is primarily driven by diffusion and not by electro-osmotic drag. However, when the current density increased to a value greater than 100 mA cm^–2^, faster water crossover was observed, and the crossover rate continued to increase at higher currents. The strong current dependence suggests that water crossover accompanies the charge-balancing ions, i.e., K^+^ and/or H^+^ cations.

Because of the water crossover, the product concentrations measured at high current densities are approximately 6.12% lower than the actual values (see detailed calculation in the *Monitoring water crossover* section of the Supporting Information), and thus the water crossover process for product quantification cannot be neglected, particularly at high current densities (≥100 mA cm^–2^).

To understand the effect of crossover on reaction stability, chronopotentiometry was performed at 200 mA cm^–2^ for longer than 6 h. As shown in Figure 5c, the reaction stopped at 5.2 h, concomitant with a drastic drop in both current and potential, and the water resonance stopped shifting toward higher values, indicating an end to the electrolyte carbonation. This is caused primarily by the continuous migration of K^+^ ions from the anode to the cathode, resulting in a reduction of the anolyte’s conductivity and ultimately the failure of the electrolyzer.

## Conclusion

We have demonstrated a new operando benchtop NMR method for studying the electrochemical reduction of CO_2_. The method was applied for copper-catalyzed eCO_2_RR. Liquid products, such as ethanol and acetate, were successfully quantified as a function of time at high current densities up to a few hundred mA cm^–2^. A rapid decrease in the Faradaic efficiencies of formate and ethanol within 5 h of reaction was observed, revealing the stability issues of the reaction at high currents.

The chemical shift of water was found to be an indicator of the CO_3_^2–^/HCO_3_^–^ concentration in the electrolyte, providing a new and likely the only known operando method to monitor the carbonation of the electrolyte solution in real time. In the 1 M KOH electrolyte, OH^–^ was converted to HCO_3_^–^ in the initial half-hour, accompanied by a pH decrease from 14 to 8. The concentration of HCO^3–^ continued to increase, which was more pronounced at higher current densities.

Water crossover rates at different currents were determined by a deuteration NMR technique and showed a strong current dependence—faster crossover at higher currents. This current dependence suggests that water crossover is, at least in part, driven by electromigration. If the current density is >100 mA/cm^2^, normally in the range of a flow cell, water crossover cannot be neglected. If it is lower than 100 mA/cm^2^, in the range of an H-cell, the water crossover driven by electro-osmotic drag is only 0.02 mL/h. In this case, the impact on the overall performance is minimal.

The NMR study revealed the highly dynamic nature of copper-catalyzed eCO_2_RR at high currents. Building upon the time-resolved observations of product selectivity, pH values, salt concentration, and water crossover, strategies such as pulsing electrolysis, carbon coating, or changing the electrolyte during the reaction can be applied, and the effects can be studied in real time for the future.

The unique combination of a gas diffusion flow cell and an NMR system via flow offers the best of both worlds, i.e., optimized electrochemical performance and ideal NMR measurement conditions. Demonstrating on a benchtop system, this operando approach is accessible to non-NMR experts and readily applicable to a wide range of catalysts, electrolyte compositions, and reactor designs for electrochemical CO_2_ reduction, and it will aid in the design and optimization of the reaction. Beyond CO_2_ reduction, the capability to capture and quantify the carbonate concentration of the electrolyte could find applications in various CO_2_ capture and CO electrolysis systems.

## Experimental Section

### Material

Phosphoric acid (ACS reagent, ≥85 wt % in H_2_O), KHCO_3_ (ACS reagent, 99.7%), KOH (ACS reagent, 85%), copper mesh (0.25 mm in thickness, 99.995%), platinum foil (0.025 mm in thickness, 99.95%), D_2_O (99.9 atom % D), and 3-(Trimethylsilyl) propionic-2,2,3,3-d4 acid sodium salt (99 atom % D) were purchased from Sigma-Aldrich. The gas diffusion layer Sigracet 39BB and proton exchange membranes Nafion 117 and Nafion 212 were purchased from the Fuel Cell store. CO_2_ gas (>99.7%) was supplied by the university central facility. The backpressure gas regulator (JR-BPR1) was obtained from VICI Jour.

### Catalyst Preparation

When quantifying formate, a Cu mesh was used as the catalyst. Before the reaction, all Cu meshes were slightly polished on both sides with 400-grit sandpaper. Subsequently, they were rinsed with demi water and dried using a stream of N_2_. The electropolishing step of the Cu mesh was performed in concentrated phosphoric acid. This was performed in a one-compartment electrochemical cell with a two-electrode setup at a potential of 1.5 V for 5 min. Another piece of Cu mesh was used as a counter electrode. Next, the electropolished Cu mesh was rinsed with demi water for a few minutes and dried with a stream of N_2_.

For all of the other experiments, Cu was deposited via Magnetron sputtering onto a laminated polytetrafluorethylene (PTFE) membrane with a polypropene backbone (0.2 μm pore size with 25 μm layer thickness, Sterlitech). The sputtering was performed using magnetic sputtering at 3 μbar of argon pressure with different sputtering times and/or sputter gun power to obtain the desired thickness. The nominal thickness of the deposited Cu was set at 300 nm.

### Electrochemical Measurements

Electrochemical measurements were performed in a three-compartment flow cell (Figure 1). Copper mesh and sputtered Cu were used as the working electrodes (cathode), Ag/AgCl was used as the reference electrode, and Pt foil was used as the counter electrode (anode). A Nafion 212 membrane separated the anode and cathode compartments to prevent crossover of anionic products and suppress the convective flow of dissolved Pt species to the cathode. Additionally, Pt is known to catalyze hydrogen evolution reaction and can be poisoned by the CO produced on the copper cathode.^33^ However, any Pt would be deposited on the electrolyte side of the Cu electrode rather than the gas-diffusion side where eCO_2_RR occurs.^13^ Therefore, the influence of the Pt counter electrode on the system is expected to be minimal. A Gamry potentiostat was used for all electrochemical measurements. Chronopotentiometry and chronoamperometry were performed on each catalyst for eCO_2_RR.

### Operando Benchtop NMR Setup

For experiments involving the quantification of ethanol, monitoring of carbonate concentration, and water crossover, a commercial NMR flow tube compatible with the Fourier 80 NMR system was used. The electrolyte solution flows from the bottom to the top of the tube. The inlet and outlet of the sampling tube were connected to two 1/16 in. PFA tubes. The PFA tube at the bottom is connected to the outlet of the electrolyte reservoir from the cathode side; the PFA tube at the top is connected to the inlet of the cathode side. The electrolyte is pumped through the sampling tube and the flow cell, which is positioned next to Fourier 80. The volume of the tubing and NMR probe is approximately 5.8 mL. At a flow rate of 2.5 mL/min, the electrolyte takes 2.3 min to flow back to the reservoir, so the time lag between the electrochemical cycling and the NMR detection is 2.3 min. Magritek 43 MHz Spinsolve was used in the experiment for quantifying formate. The electrochemical setup is the same, while a flow sampling tube (Kit RM2) from Magritek was used.

### NMR Parameters

Bruker Fourier 80 was used to monitor the liquid products and the water crossover process. Observing alcoholic products on a benchtop NMR is a nontrivial task because the resonance frequency difference between, e.g., the methyl group of ethanol and solvent water is only 275 Hz, in contrast to a much larger difference of 2062 Hz on a conventional 600 MHz NMR system. A water suppression NMR pulse sequence WATERGATE 3919 needs to be carefully optimized to suppress the water resonance while avoiding oversuppressing the ethanol resonances (see Figure S12 for pulse sequence parameters).

Applying the WATERGATE 3919 pulse sequence, ^1^H NMR spectra were acquired. The number of scans was 32 with a recycle delay of 11.3 s, which was higher than three times the longitudinal relaxation time *T*_1_, where the *T*_1_ of CH_3_ protons in ethanol was measured to be 3.7 s. For flow experiments, a sufficiently long residence time on the order of T_1_ in the detection region should be set to allow the buildup of magnetization. At a flow rate of 2.5 mL/min and for an effective detection volume of 0.396 mL, the residence time is 9.5 s. To enhance the signal-to-noise ratio, seven spectra were sequentially added together. For monitoring water crossover, a single pulse sequence with 1 scan and a recycle delay of 34 s was used.

Magritek 43 MHz Spinsolve was used to quantify formate. The products were measured using 32 scans, with an acquisition time of 3.2 s per scan and a recycle delay of 10 s. A quick shim protocol was performed after each measurement to keep the system in an optimal configuration during the whole reaction monitoring process.

### Formate and Ethanol Quantification

For formate, 0.01, 0.1, 0.5, and 1 M potassium formate solutions were prepared. These standards flowed into a Magritek 43 MHz Spinsolve using the same flow rate of 2.5 mL/min. The peak areas for each standard were measured and used to construct a calibration curve, which can be found in Figure S1b.

In the experiment to quantify ethanol, three standard samples with concentrations of 0.001, 0.01, and 0.1 M were flowed into the Fourier 80 system at a flow rate of 2.5 mL/min. The CH_3_ signal was integrated to establish a calibration curve, as shown in Figure S6.

#### Partial Current Density Calculation

The partial current density of formate can be calculated using the formula below:

$$j_{\mathrm{formate}}=FE_{\mathrm{formate}}\times j_{\mathrm{total}}$$

*j*_formate_: partial current density of formate

FE_formate_: faradaic efficiency of formate

*j*_total_: total current density

With the concentration profile of formate tracked over time, the time-dependent FE for formate can be calculated using the equation below:

$$FE_{\mathrm{formate}}=\frac{zcVF}{\int_{0}^{t}Itdt}$$

*z*: number of charge transferred to form formate

*c*: concentration of formate obtained from NMR quantification

*V*: volume of the electrolyte

*F*: Faradaic constant

## Supplementary Material



## Notes

Additional text, figures, and tables are organized into seven sections: 1. Operando NMR quantification of formate; 2. quantification of C_2_^+^ liquid products; 3. monitoring ethanol crossover; 4. measuring pH as a function of time; 5. quantifying time-resolved bicarbonate concentration; 6. monitoring water crossover; 7. NMR water-suppression pulse sequence (PDF)
