# Supplementary material for: Operando Benchtop NMR Quantifies Carbonation, Water Crossover, and Liquid Products for High-Current Electrochemical CO2 Reduction
Source: ACS Catal. 2025 Jul 7;15(14):12300–7. doi: 10.1021/acscatal.5c00355 (PMC12281561; doi:10.1021/acscatal.5c00355)
Supplement: Supplementary file 1 [file cs5c00355_si_001.pdf]

Operando benchtop NMR quantifies carbonation, water crossover and liquid products for high-current electrochemical CO_2 reduction

Zhiyu Zhu,[1] Kaan Zeki Çolakhasanoĝlu,[1] Ruud L.E.G. Aspers,[1] Joris Meurs,[2] Simona Cristescu,[2] Thomas Burdyny,[3] Evan Wenbo Zhao[1]*

[1]Magnetic Resonance Research Center, Institute for Molecules and Materials, Radboud University, 6525 AJ Nijmegen, the Netherlands

[2]Life Science Trace Detection Laboratory, Institute for Molecules and Materials, Radboud University, 6525 AJ Nijmegen, Netherlands

[3]Department of Chemical Engineering, Delft University of Technology, 2629 HZ Delft, the Netherlands

*Correspondence email: evanwenbo.zhao@ru.nl

Table of Contents

1. Operando NMR quantification of formate 1

2. Quantification of C_2^+ liquid products 4

3. Monitoring ethanol crossover 7

4. Measuring pH as a function of time 8

5. Quantifying time-resolved bicarbonate concentration..10

6. Monitoring water crossover 11

7. NMR water-suppression pulse sequence 13

1. Operando NMR quantification of formate

Magritek 43 MHz Spinsolve was used to quantify the concentration of formate during eCO_2RR. The ^1H spectra were acquired with 32 scans and an acquisition time of 3.2 s for each scan. The internal standard trimethylsilylpropanoic acid (TSP) is at 0 ppm. The electrolyte flow rate was 2.5 mL/min. Fig. S1a displays a slice of ^1H NMR spectrum from the operando experiments. To quantify the formate concentration, we prepared 0.01 M, 0.1 M, 0.5 M and 1 M potassium formate solutions. These standard solutions flowed into Magritek 43 MHz Spinsolve at the same flow rate of 2.5 mL/min. The signal for each standard were integrated and used to construct a calibration curve, as presented in Fig. S1b.

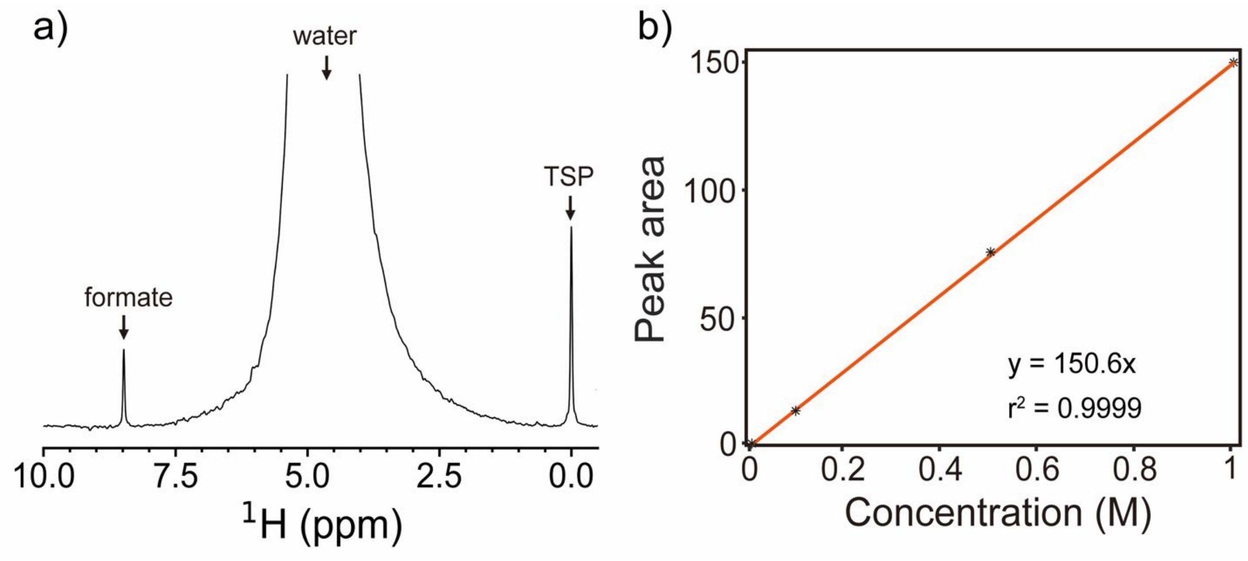

Fig. S1. (a) A representative ^1H NMR spectrum during eCO_2RR. (b) A calibration curve for formate quantification.

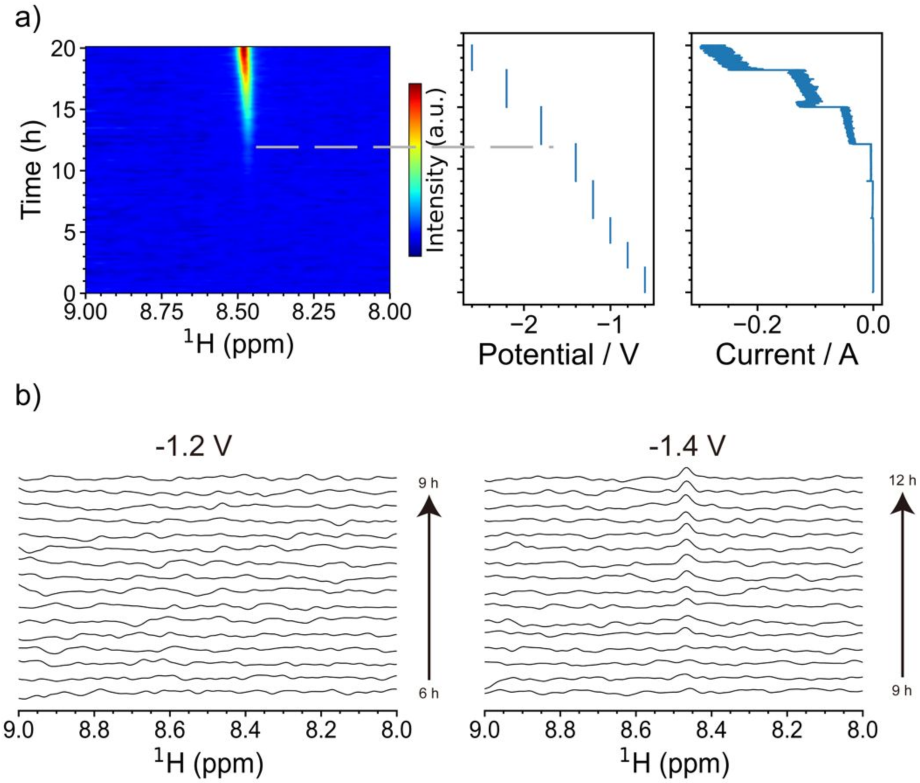

Fig. S2. (a) ^{1}H NMR contour plot showing the evolution of the formate resonances as a function of time (left) and the corresponding potential and current profile (right). The grey dashed line highlights the starting point of significant formate formation. (b) Stacked ^{1}H NMR spectra obtained at -1.2 V and -1.4 V, respectively. At -1.2 V, there are no detectable formate signals. Formate signal at 8.45 ppm started to emerge at -1.4 V.

Calculation of FE for formate as a function of potential

According to the Faraday's law of electrolysis, the FE can be calculated using the following formula:

$$FE = \frac{zcVF}{\int_0^t It \, \mathrm{dt}}$$

z: charge transfer of formate

c: concentration of formate obtained from NMR quantification

V: volume of the electrolyte

F: Faradaic constant

Table S1: the overall FE at different potentials.

Potential	Charge to formate	$\int_0^t It$	FE
-1.4 V	3.09 C	53.96 C	5.72 %
-1.8 V	10.03 C	249.1 C	4.03 %
-2.2 V	15.82 C	739.41 C	2.14 %
-2.6 V	21.23 C	1665.8 C	1.27 %

2. Quantification of C_2^+ liquid products

To observe the C_2^+ liquid products in H_2O, a water suppression pulse sequence was required. In Fig. S3. we compared several solvent suppression pulse sequences on Fourier 80 benchtop NMR using ethanol as the test sample.[1,2] Because the ethanol peak is very close to the water peak on Fourier 80 benchtop NMR, the intensity of the CH_2 peak is inevitably affected by the pre-saturation pulse sequence. The watergate w3 pulse sequence provides better results. Although watergate w5 suppresses more water signals, its longer binomial sequence induced more severe phase distortion.[2,3] As a result, the watergate w3 pulse sequence was selected in our experiment.

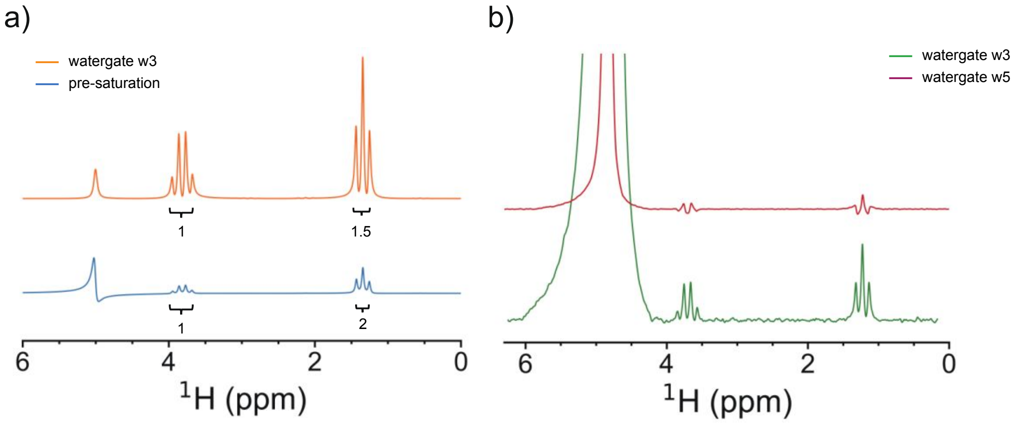

Fig. S3. (a) ^{1}H NMR spectra of 3 M ethanol dissolved in H_2O using watergate w3 and pre-saturation pulse sequences. (b) ^{1}H NMR spectra of 1 mM ethanol using watergate w3 and watergate w5 pulse sequences.

The sputtered Cu catalyst was used to increase the selectivity for ethanol. Chronopotentiometry was performed at 100 mA/cm^2 while ^{1}H NMR spectra were acquired simultaneously. As shown in Fig. S4a, the potential first decreased from –2.3 V to –3.2 V vs. Ag/AgCl and then gradually increased to –2.5 V over time. In the operando ^{1}H NMR spectra (Fig. S4a), the formate signal appears as a singlet at 8.5 ppm, the CH_3 group of ethanol as a triplet at 1.2 ppm, the CH_2 group of ethanol as a quartet at 3.7 ppm, and acetate as a singlet at 1.9 ppm. A weak signal at 0.9 ppm is

from n-propanol, becoming more pronounced in the spectrum collected with more accumulation of scans (Fig. S5). The internal standard TSP is at 0 ppm.

For the ethanol signals, the intensity initially increases and then gradually decreases, as shown in Fig. S4a. During the decrease, no new signals were observed, so we ruled out the possibility of further reaction. We attributed it to the degradation of the catalytic activity and dilution of the solution by water, the latter is systematically studied and reported in the section *Monitoring water crossover* in the main matter and the ethanol crossover to the other compartment, verified in Fig. S7. To obtain a better signal to noise ratio of the products, we collect the electrolyte after the reaction and use benchtop NMR to measure it with a high number of scans of 2000. The spectrum is presented in Fig. S5. The triplet next to the ethanol resonance is assigned to the n-propanol.

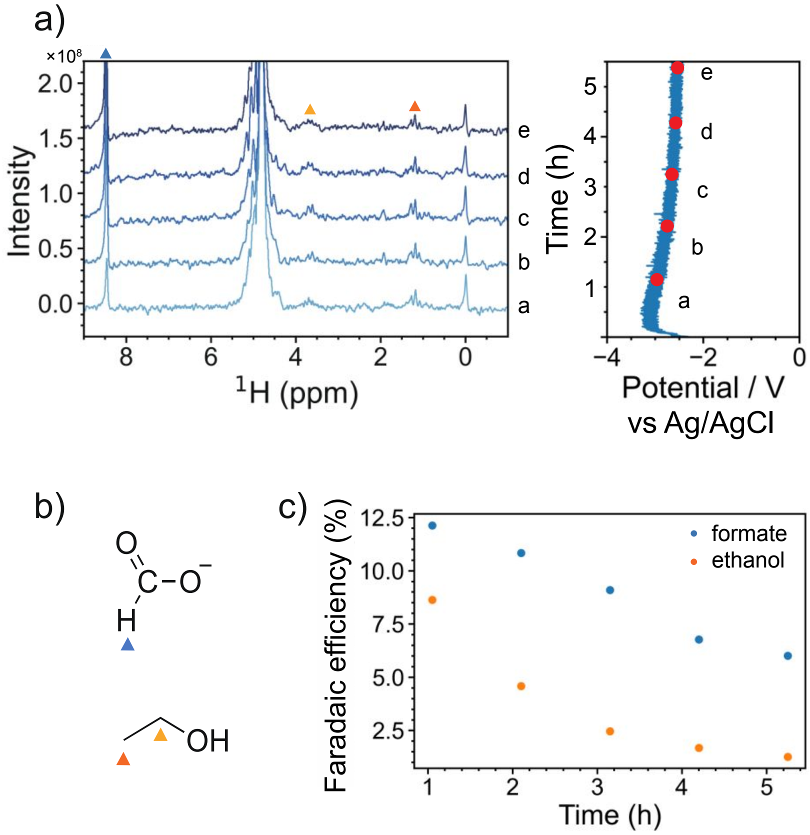

Fig. S4. (a) ^{1}H NMR as a function of time (left) and the corresponding potential profile (right). Every spectrum represents the time-averaged result for 1 h, which is marked as a red dot. (b) The molecular structure of the liquid products, formate and ethanol. (c) FE of formate and ethanol during the reaction. The CH_3 signal at 1.2 ppm of ethanol was used for the quantification.

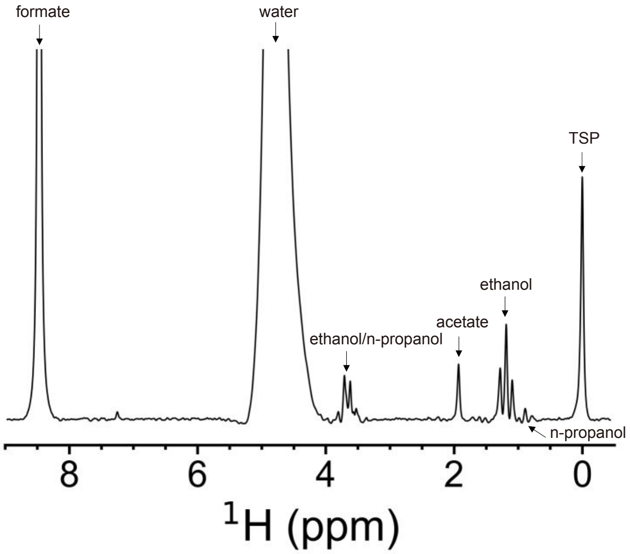

Fig. S5. ^{1}H NMR spectrum obtained on benchtop NMR with water suppression after the reaction.

Calculation of time-resolved FE

The concentration of ethanol was calculated by using a calibration curve, which is presented in Fig. S6. Three samples with concentrations of 0.001 M, 0.01 M and 0.1 M are prepared, and signals from CH_3 were used for the integration.

The concentration of formate can be further calculated according to the ethanol concentration.

$$c = \frac{S}{S_s} \times \frac{N_s}{N} \times c_s$$

where c is the concentration, S is the signal area, and N is the number of protons. The subscript *s* stands for standard, that is, ethanol for this experiment.

Thus, time-resolved FEs can be calculated and are presented in the following table.

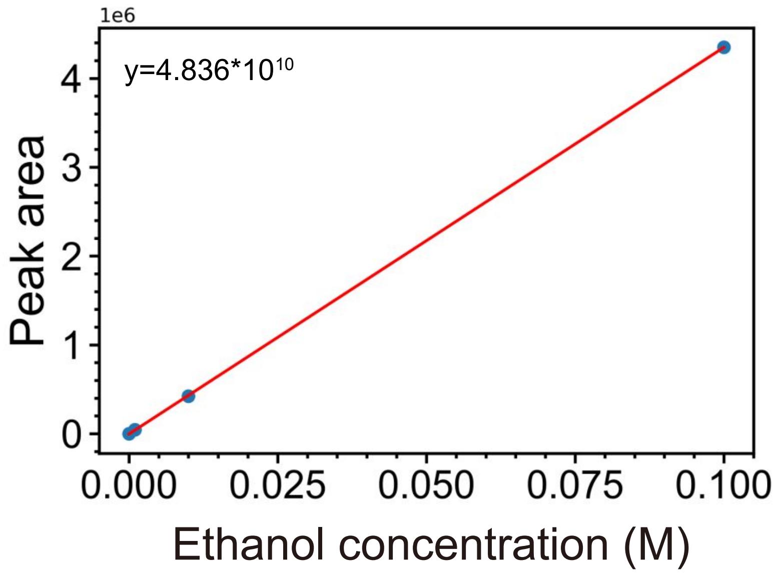

Fig. S6. Calibration curve of ethanol.

Table S2: time-resolved FE of formate.

Formate peak area	Conc. (M)	Charge to formate (C)	$\int_0^t Itdt$ (C)	FE
382956418.32	0.008206	45.8425	378 ()	0.121
684851643.33	0.014674	81.9814	756	0.108
861759805.06	0.018465	103.1586	1134	0.091
856501007.57	0.018352	102.5291	1512	0.068
949594599.012	0.020347	113.6730	1890	0.0601

Table S3: time-resolved FE of ethanol.

Ethanol peak area	Conc. (M)	Charge to ethanol (C)	$\int_0^t Itdt$ (C)	FE
272916086.05	0.0056	32.6689	378	0.0864
289947000.55	0.0060	34.7076	756	0.0459
233575662.47	0.0048	27.9598	1134	0.0247
212891590.20	0.0044	25.4838	1512	0.0169
200975883.26	0.0042	24.0575	1890	0.0127

3. Monitoring ethanol crossover

To verify ethanol crossover, the same flow setup was employed using 10 mL 0.01 M ethanol solution as the catholyte and 10 mL pure water as the anolyte, separated by a Nafion 212 membrane. Anolyte flowed to the benchtop NMR at a flow rate of 2.5 mL/min. By monitoring the signal of the anolyte as a function of time, without applying a current or potential, the ethanol signals were detected and grew in intensity, confirming ethanol crossover through the Nafion membrane.

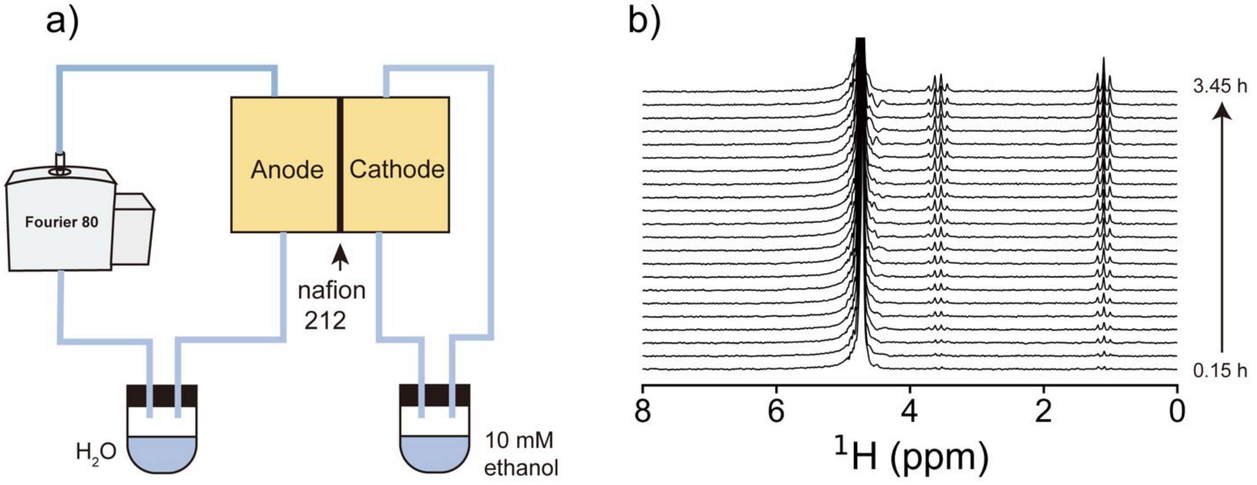

Fig. S7. (a) Schematic of the setup for measuring ethanol crossover. (b) ^{1}H NMR spectra of the anolyte as a function of time.

4. Measuring pH as a function of time

The electrochemical potentials corresponding to the spectra shown in Fig. 3 are presented in Fig. S8. The fluctuation of the potential is caused by the bubble formation from gaseous products. We measured the pH values during the reaction. The results are presented in Fig. S9.

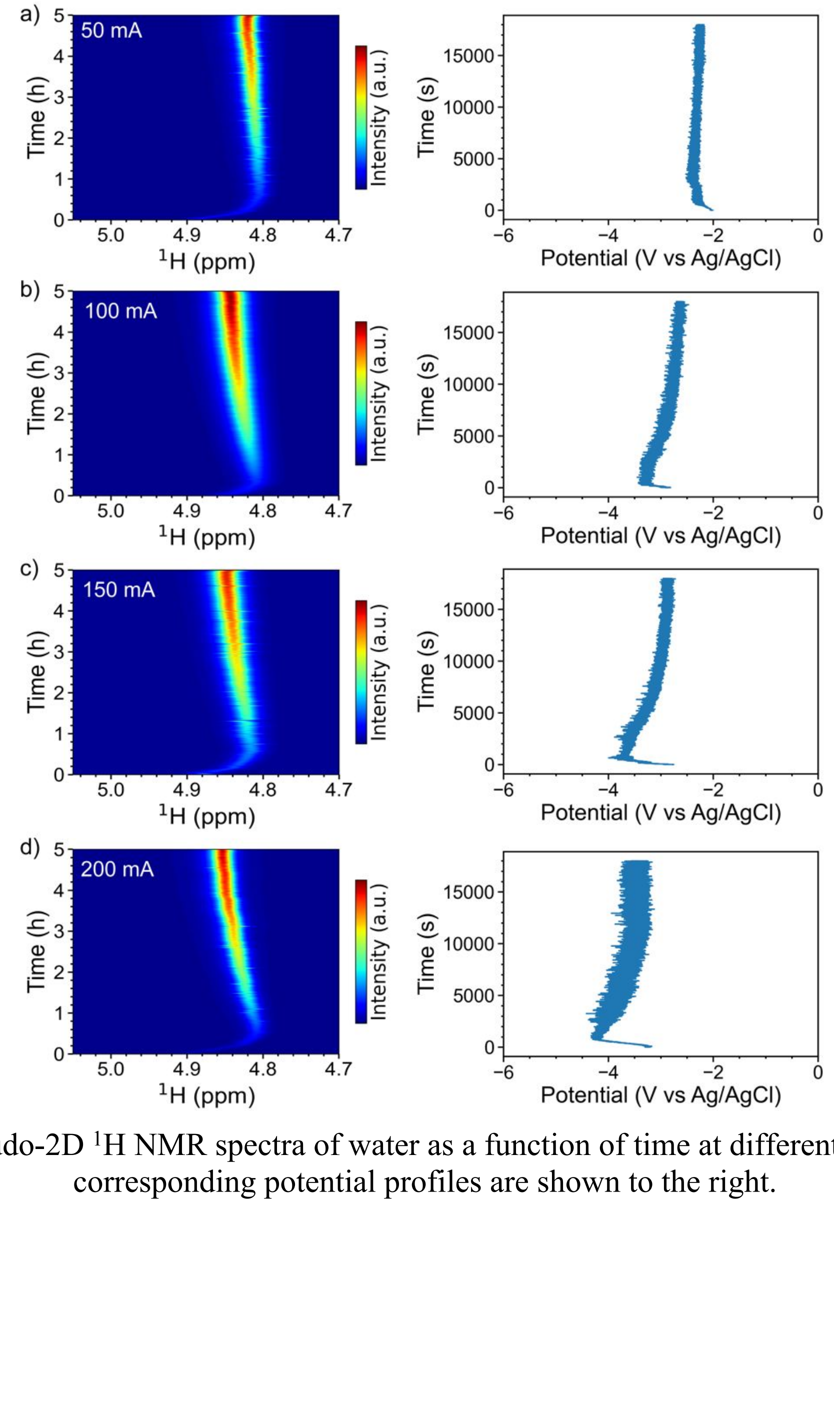

Fig. S8. Pseudo-2D ^{1}H NMR spectra of water as a function of time at different currents. The corresponding potential profiles are shown to the right.

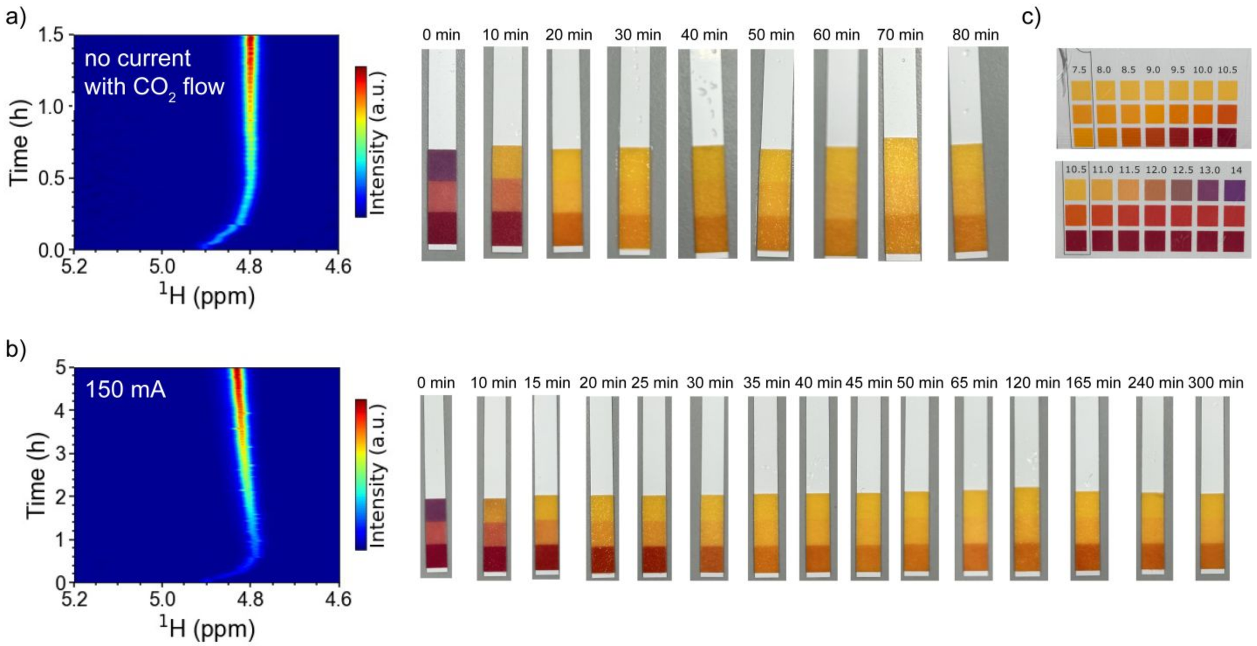

Fig. S9. (a-b) Pseudo-2D ^1H NMR spectra of water as a function of time without a current and at 150 mA, respectively. The pH papers used for measuring the pH values are shown to the right. (c) Reference for the pH measurements.

5. Quantifying time-resolved bicarbonate concentration

We obtained the $KHCO_3$ and K_2CO_3 calibration curve, which is presented in Fig. 4c. The concentration and ^1H chemical shift relationships were derived as follows:

$$\delta(H_2O) = 0.015[HCO_3^-] + 4.8$$

$$\delta(H_2O) = 0.068[CO_3^{2-}] + 4.8$$

The equilibrium between HCO_3^- and CO_3^{2-} is

$$CO_3^{2-} + H^+ \rightleftharpoons HCO_3^-, \qquad pK_a = 10.3$$

Which can be converted to

$$\frac{[HCO_3^-]}{[CO_3^{2-}]} = 10^{pK_a - pH}$$

In the electrolyte, the chemical shift of water has a relationship to the (bi)carbonate concentration:

$$\delta(H_2O) = 0.015[HCO_3^-] \times \frac{[HCO_3^-]}{[CO_3^{2-}] + [HCO_3^-]} + 0.068[CO_3^{2-}] \times \frac{[CO_3^{2-}]}{[CO_3^{2-}] + [HCO_3^-]} + 4.8$$

Thus, the bicarbonate concentration can be calculated according to:

$$[HCO_3^-] = \frac{\delta(H_2O) - 4.8}{0.068 + 0.015 \times (10^{pK_a - pH})^2} \times 10^{pK_a - pH} \times (10^{pK_a - pH} + 1)$$

The calculated bicarbonate concentrations during the operando NMR experiments at different currents are presented below. Considering the errors from the pH measurements, the bicarbonate concentrations were calculated for pH values of 7.5, 8, 8.5, 9 and 9.5. Negligible differences were obtained since in this pH range, bicarbonate is the dominant anionic species. The chemical shift of water was corrected according to the corresponding TSP chemical shift.

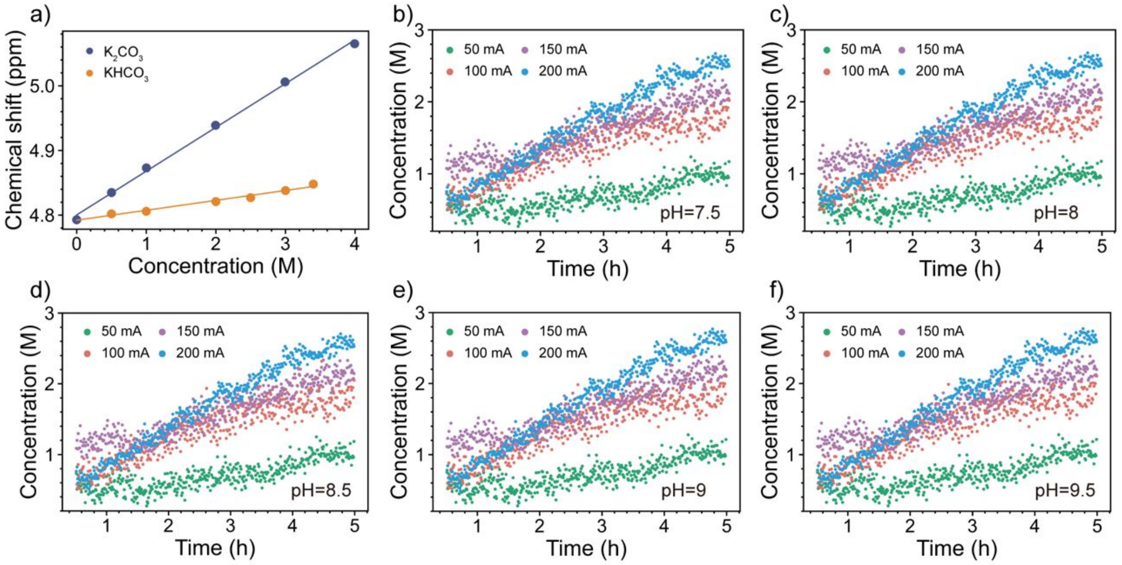

Fig. S10. (a) ^{1}H chemical shift of water as a function of $KHCO_3$ and K_2CO_3 concentrations. (b-f) HCO_3^- concentrations calculated for a pH range from 7.5 to 9.5.

6. Monitoring water crossover

At the anode, water oxidation reaction happened.

$$2H_2O - 4e^- \rightarrow O_2 + 4H^+$$

The generation of H^+ results in the acidification of the anolyte, which might lead to a change of the main charge carrier from K^+ to H^+. Thus, we calculate the produced H^+ concentration by Faraday's laws of electrolysis:

$$n_{H^+} = \frac{It}{F}$$

where I is the current, t is the total time, and F is the Faraday constant. At 200 mA for 5 hours electrolysis, the n_{H^+} is 0.037 mol, which is smaller than the n_{OH^-} of 0.05 mol at the anode. As a result, we assume K^+ is the main charge carrier during all experiments.

To calculate the H_2O content during the reaction, we prepared solutions with different concentrations of H_2O in D_2O of 0%, 25%, 50%, 75%, and 100%. Each solution was flowed into the benchtop NMR at a flow rate of 2.5 mL/min. The peak areas were measured and used to construct a calibration curve as presented below.

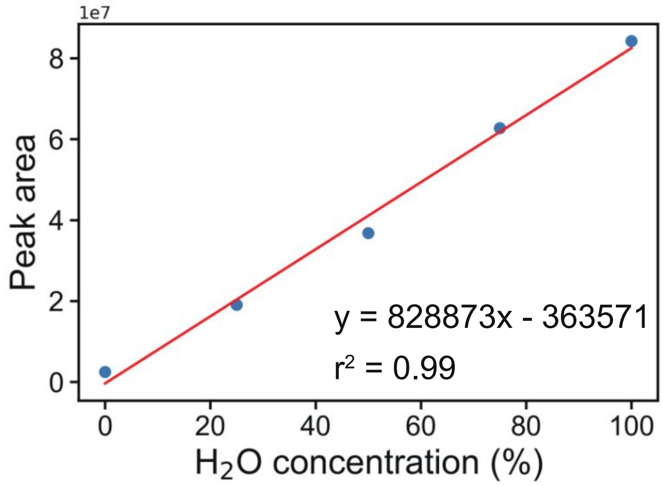

Fig. S11. Calibration curve of H_2O concentration.

When there is no current, the water crossover rate is 0.448 mL/h. At 100 mA, the water crossover rate is 1.1 mL/h. Thus, the water crossover rate caused by the current is 0.652 mL/h. Because the catholyte volume is 10 mL, the resulting error over one hour of reaction can be estimated:

$$\frac{\frac{1}{10} - \frac{1}{10.652}}{\frac{1}{10}} \times 100\% = 6.12\%$$

7. NMR water-suppression pulse sequence

a)

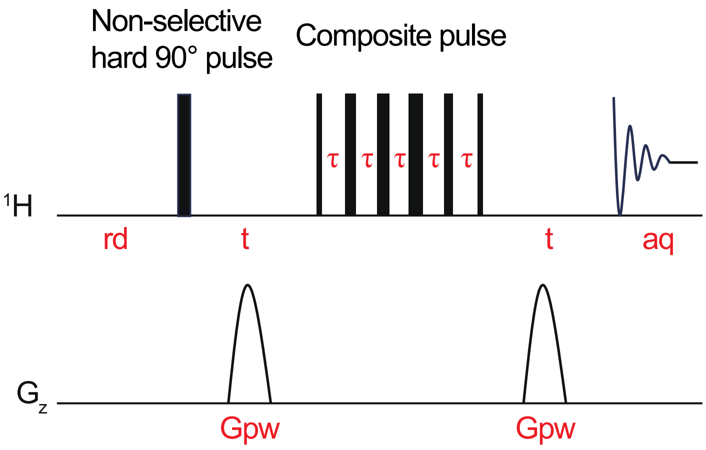

b)

PLW	90°	Gpw	t	τ	aq	rd
1.03 W	11.71 μs	1000 μs	0.00025 s	0.0011 s	6 s	11.3 s

Fig. S12. (a) The pulse sequence of Watergate w3. It consists of a non-selective hard 90° pulse, a composite pulse (consisting of 6 hard pulses separated by a delay τ), and two gradient pulses. The gradient pulse dephases all the resonances. The composite pulse acts as a 180° pulse for all frequencies except those on resonance with water and multiples of n/τ away from the transmitter. Spectral width between two null points equals to 1/τ. Thus, the suppression bandwidth narrows as the binomial delay is lengthened, with a 30% non-inversion region for w3. (b) The parameters used in NMR measurements. The power (PLW) on the ^{1}H channel is 1.03 W. The 90° pulse has a pulse width of 11.71μs, followed by a gradient pulse with a pulse width (Gpw) of 1000 μs and a time delay (t) of 0.00025 s. The time delay within the composite pulse (τ) is 0.0011 s. The data acquisition time (aq) is 6 s, and the recycle delay (rd) is 11.3 s. The shape of the gradient pulse is SMSQ10.100.

Reference

(1) Liu, M.; Mao, X.; Ye, C.; Huang, H.; Nicholson, J. K.; Lindon, J. C. Improved WATERGATE Pulse Sequences for Solvent Suppression in NMR Spectroscopy. *Journal of Magnetic Resonance* **1998**, *132* (1), 125–129. https://doi.org/10.1006/jmre.1998.1405.

(2) Soong, R.; Wolff, W.; Pellizzari, J.; Downey, K.; Chen, S.; Biswas, R. G.; Bastawrous, M.; Goerling, B.; Busse, V.; Busse, F.; Elliott, C.; Haber, A.; Belguise, A.; Simpson, M.; Simpson, A. Water Suppression 101 for Benchtop NMR–An Accessible Guide and Primer Including Fully Interactive Training Videos. *Journal of Magnetic Resonance Open* **2024**, *19*, 100150. https://doi.org/10.1016/j.jmro.2024.100150.

(3) Wang, J.; Zhang, X.; Sun, P.; Jiang, X.; Jiang, B.; Cao, C.; Liu, M. The Impact of Pulse Duration on Composite WATERGATE Pulse. *Journal of Magnetic Resonance* **2010**, *206* (2), 205–209. https://doi.org/10.1016/j.jmr.2010.07.007.
